# Tirzepatide: A Promising Drug for Type 2 Diabetes and Beyond

**DOI:** 10.7759/cureus.38379

**Published:** 2023-05-01

**Authors:** Palak Dutta, Yashaank Kumar, Alexis T Babu, Suganya Giri Ravindran, Ajal Salam, Bhumish Rai, Aakash Baskar, Ananya Dhawan, Manjima Jomy

**Affiliations:** 1 General Medicine, Kyiv Medical University, Kyiv, UKR; 2 Medicine and Surgery, Soochow University, Suzhou, CHN; 3 Medicine, Tbilisi State Medical University, Tbilisi, GEO; 4 Internal Medicine, California Institute of Behavioral Neurosciences & Psychology, Fairfield, USA; 5 Medicine and Surgery, Government Medical College, Kottayam, IND; 6 Medicine and Surgery, Pandit Bhagwat Dayal Sharma Post Graduate Institute of Medical Sciences (PGIMS), Rohtak, IND; 7 Medicine and Surgery, K.A.P. Viswanatham Government Medical College, Tiruchirappalli, IND; 8 Medicine and Surgery, Southeast University, Nanjing, CHN

**Keywords:** meta-analysis, surmount, surpass, obesity, glp-1, tirzepatide, diabetes, gip

## Abstract

Tirzepatide is a promising drug with dual-acting glucose-dependent insulinotropic polypeptide (GIP) and glucagon-like peptide 1 (GLP-1) receptor activation that has revolutionized the treatment of type 2 diabetes mellitus (T2DM) as an adjunct to diet and exercise. In phase 3 clinical trials (SURPASS 1-5), the dose-dependent efficacy and safety of tirzepatide were assessed by once-weekly subcutaneous injection (5 mg, 10 mg, and 15 mg), as monotherapy or combination therapy, in individuals with T2DM. Tirzepatide has been shown to achieve better glycemic control in terms of glycosylated hemoglobin reduction and improved fasting and postprandial glucose levels as compared to other diabetic medications. Moreover, the studies demonstrate a reduction in body weight (-6.2 to -12.9 kg), and other cardiovascular benefits by altering the lipid profile, reducing blood pressure, and visceral adiposity. Tirzepatide has acceptable side effects and is well tolerated, with a low risk of hypoglycemia. The SURPASS 4 clinical trial has shown positive cardiovascular outcomes in people with T2DM and elevated cardiovascular risk. Additionally, encouraging results from SURMOUNT trials and ongoing SURPASS-CVOT studies will shed more light on cardiovascular safety in the future. In this review, we have summarized the clinical trials and their respective outcomes and highlighted the potential future indications for tirzepatide in the management of obesity, heart failure, and nonalcoholic steatohepatitis.

## Introduction and background

Tirzepatide is a novel diabetes medication that has been approved by the Food and Drug Administration (FDA) [1]. It is the first of its kind to act as a dual-acting glucose-dependent insulinotropic polypeptide (GIP) and glucagon-like peptide 1 (GLP-1) receptor agonist [2,3]. Finan et al. coined the term "twincretin" to describe their synergistic effect on insulin secretion [2,3]. After 15-20 minutes of meal ingestion, plasma concentrations of GLP-1 and GIP increase, stimulating their receptors on pancreatic cells to activate an insulinotropic response that is glucose-dependent and proportional to facilitate the elimination of the absorbed carbohydrate and fat load [4,5]. Tirzepatide is a viable alternative for supplementing the incretin effect in achieving glycemic control as this impact is weakened in diabetes, with the added benefits of glycosylated hemoglobin (HbA1c) reduction, weight loss, cardiovascular health, a good lipoprotein profile, and improvement in nonalcoholic steatohepatitis (NASH) [6,7]. In a series of clinical trials, the effectiveness of tirzepatide at varying doses has been compared with that of other currently prescribed drugs, including semaglutide, dulaglutide, insulin degludec, insulin glargine, metformin, etc. Tirzepatide significantly outperformed dulaglutide, semaglutide, degludec, and glargine in terms of its effects on postprandial and fasting blood glucose levels as well as the likelihood that patients using it would experience a decrease in HbA1c [8-10].

According to research, tirzepatide's benefits outweighed its drawbacks, making it the first of its kind to be studied. Nevertheless, given its novelty, further investigation is required to gain a comprehensive understanding of its potential adverse effects [10]. We aim to discuss tirzepatide in depth in this review, highlighting the benefits of this drug in the treatment of diabetes and other conditions such as obesity, cardiovascular safety, and liver disease. Furthermore, we compared it to other anti-diabetic medications that have been shown to be effective and safe. With the current literature available on tirzepatide, it appears to be a ray of hope for diabetic patients, stressing the critical need for additional long-term and large-scale studies.

## Review

Mechanism of action

The Role of Incretins in the Pathophysiology of Type 2 Diabetes Mellitus

Tirzepatide is a dual-acting GIP and GLP-1 agonist with a higher affinity for GIP receptors and is highly effective in not just maintaining a normal glycemic state but also for weight loss in type 2 diabetes mellitus (T2DM). Elrick et al. were the first to report significantly higher plasma insulin concentrations following oral glucose loading compared to parenteral therapy [11]. This phenomenon is referred to as the “incretin effect” and has been proven to account for as much as 65% of postprandial insulin secretion [12]. Incretin hormones encompass GLP-1 and GIP [13].

Plasma concentrations of GLP-1 and GIP are very low during fasting and rise 15-30 minutes after a meal [5]. Once produced, GIP and GLP-1 stimulate their receptors on pancreatic cells to activate an insulinotropic response that is glucose-dependent and proportionate to promote the removal of the absorbed carbohydrate and fat load [4]. Incretin effects are relatively short-lived since the hormones are active for about one to two minutes after release before being deactivated by the enzyme dipeptidyl peptidase-4 (DPP-4) [4]. The effect of incretin is tremendously lowered in T2DM individuals compared to non-diabetic individuals [13]. Reduced incretin hormone synthesis in response to meals (hyposecretion) and diminished insulinotropic action on pancreatic beta cells [14] are two explanations that have been put forward for the attenuation of the incretin effect in T2DM [15].

Mechanism of Action of Tirzepatide on Insulin and Adipose Tissue

Tirzepatide was discovered by incorporating GLP-1 activity into the GIP sequence [16]. The dietary-stimulated levels of GLP-1 are reduced in T2DM, whereas the insulinotropic effects after infusion of pharmacological levels of GLP-1 are similar in people with diabetes and those who are euglycemic [17,18]. Moreover, GLP-1 agonists also have a satiety effect [19].

The GIP receptor is expressed in white adipose tissues [20], which act as a buffer for circulating lipids [21]. GIP receptor agonism is thought to boost adipocytes' ability to quickly clear dietary triglycerides (TAG) as well as long-term lipid storage by allowing the healthy growth of white adipose tissue. GIP activity in the central nervous system may have additional metabolic advantages by lowering energy expenditure in addition to enhancing lipid management in the periphery, particularly when paired with GLP-1 [6].

**Figure 1:**
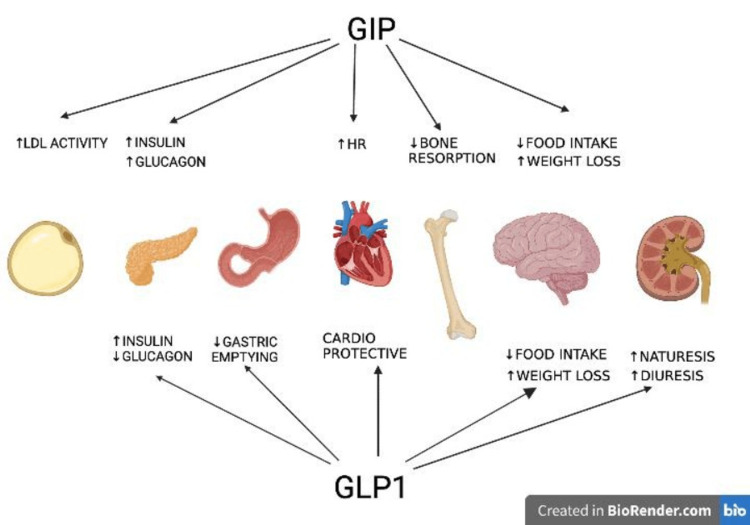
Mechanism of action of tirzepatide GIP = glucose-dependent insulinotropic polypeptide; GLP-1 = glucagon-like peptide 1; LDL = low-density lipoprotein; HR = heart rate.

In comparison to administering each hormone separately, giving healthy people GIP and a GLP-1 receptor agonist together produces a synergistic effect that increases insulin secretion [22]. Finan et al. were the first to describe the synergistic effect of GIP and GLP-1 and create a single-molecule dual agonist of GIP and GLP-1 receptors, thus coining the term "twincretin” [3]. The significant improvement in the patient's health is consistent with the theory that supplementing GLP-1 therapy with GIP pharmacology improves glucose control through the double action on the pancreatic cells, which would lead to the enhancement of insulin secretion. GIP also helps to improve the function of white adipose tissue while exerting a potent anorexigenic effect by combining the activation signals of the above two receptor pathways inside the brain [6]. Additionally, tirzepatide has demonstrated a significant impact on reducing the fasting circulating triglyceride levels [7]. Also, homeostatic model assessment for insulin resistance (HOMA2-IR) analysis indicates an increase in insulin sensitivity [23]. The mechanism of action of tirzepatide is shown in Figure 1 [24].

Overview of clinical trials

In the wake of phase I and phase II trials’ promising results, the clinical trials of phase III (Table 1) were designed to evaluate the safety and efficacy of tirzepatide for improving glycemic control in patients with T2DM in eight published SURPASS trials (SURPASS 1-5, SURPASS J-mono, and SURPASS J-combo) [8-10,25-28] and for treating obesity in the SURMOUNT trial (SURMOUNT-1) [29]. The SURPASS AP-combo [30] has concluded, but the results have not yet been released.

**Table 1:** Summary of completed randomized controlled phase III trials. HbA1C = glycated hemoglobin; BMI = body mass index; FSG = fasting serum glucose; DPP4 = dipeptidyl peptidase-4; OAM = oral antihyperglycemic medication; MACE = major adverse cardiovascular event; SAE = severe adverse event; SGLT-2 = sodium glucose cotransporter; RCT = randomized controlled trials; T2DM = type 2 diabetes mellitus; HOMA = homeostasis model assessment; HDL = high density lipoprotein; VLDL = very low density lipoprotein; LDL = low density lipoprotein; NCT = National Clinical Trial; SC = subcutaneous

Study, NCT number	Sample size, duration, design	Participant(s)	Agent(s) intervention(s)	Primary outcome(s)	Secondary outcome(s)
SURPASS-1 [25] (NCT03954834)	N = 478, 40 weeks, double-blind placebo-controlled	T2DM (≥18 years old), naive to diabetes injectable therapies, ≥3 months since taking OAM, ≥3 months stable body weight, HbA1C 7.0% to 9.5%, BMI ≥ 23 kg/m2	Agents: weekly SC dose of 5 mg tirzepatide (n = 121), 10 mg tirzepatide (n = 121), 15 mg tirzepatide (n = 121); intervention: SC placebo (n = 115)	The HbA1C mean change from precedent (5 mg) -1.75%, (10 mg) -1.71%, (15 mg) -1.69%, (placebo) -0.09%	Body weight changes from precedent (5 mg) -6.3 kg, (10 mg) -7.0 kg, (15 mg) -7.8 kg, (placebo) -1.0 kg. FSG changes from precedent (5 mg) -39.6 mg/dL, (10 mg) -39.8 mg/dL, (15 mg) -38.6 mg/dL, (placebo) +3.7 mg/dL. Fasting lipids change from precedent cholesterol (15 mg) -8.4%, triglycerides -21%, LDL -12.4%, VLDL -19.8%, HDL +7.5%
SURPASS-2 [8] (NCT03987919)	N = 1,879, 40 weeks, open-label	T2DM, mean age = 56.6 years, HbA1C = 7.0% to 10.5%, treatment with metformin for ≥3 months (≥1,500 mg per day), stable body weight ≥3 months, BMI of ≥25 kg/m2	Agents: weekly SC dose of 5 mg tirzepatide (n = 470), 10 mg tirzepatide (n = 469), 15 mg tirzepatide (n = 470); intervention: weekly SC dose of semaglutide (n = 469)	The HbA1C mean change from precedent (5 mg) -2.01%, (10 mg) -2.24%, (15 mg) -2.30%, (semaglutide) -1.86%	Body weight changes from precedent (5 mg) -7.6 kg, (10 mg) -9.3 kg, (15 mg) -11.2 kg, (semaglutide) -5.7 kg. Hypoglycemia less than 54 mg/dL in (5 mg) 0.6%, (10 mg) 0.2%, (15 mg) 1.7%, (semaglutide) 0.4% Fasting lipids change from precedent (15 mg) triglycerides -24.8%, VLDL -23.7%, HDL +7.1%
SURPASS-3 [8] (NCT03882970)	N = 1,444, 52 weeks, open-label	T2DM, insulin naive, HbA1C = 7.0% to 10.5%, stable metformin (≥1,500 mg/day) or metformin and SGLT-2 inhibitor treatment ≥3 months, stable body weight ≥3 months, BMI of ≥25 kg/m2	Agents: weekly SC dose of 5 mg tirzepatide (n = 358), 10 mg tirzepatide (n = 360), 15 mg tirzepatide (n = 359); intervention: daily SC dose of insulin degludec (n = 360)	The HbA1C mean change from precedent (5 mg) -1.85%, (10 mg) -2.01%, (15 mg) -2.14%, (insulin degludec) -1.25%	Body weight changes from precedent (5 mg) -7.0 kg, (10 mg) -9.6 kg, (15 mg) -11.3 kg, (insulin degludec) +1.9 kg. Hypoglycemia less than 54 mg/dL in (5 mg) 1.4%, (10 mg) 1.1%, (15 mg) 2.2%, (insulin degludec) 7.3%
SURPASS-4 [10] (NCT03730662)	N = 2,002, 104 weeks, open-label	T2DM, HbA1C = 7.5% to 10.5%, ≥1-month stable treatment, ≤3 oral antihyperglycemic drugs (metformin, SGLT-2 inhibitors, sulfonylureas), ≥3 months stable treatment, ≥3 months stable body weight, BMI ≥ 25 kg/m2, increased risk of cardiovascular events	Agents: weekly SC dose of 5 mg tirzepatide (n = 329), 10 mg tirzepatide (n = 328), 15 mg tirzepatide (n = 338); intervention: daily SC dose of insulin glargine (n = 1,000)	HbA1C mean change from precedent (5 mg) -2.11%, (10 mg) -2.30%, (15 mg) -2.41%, (insulin glargine) -1.39%	Body weight changes from precedent (5 mg) -6.4 kg, (10 mg) -8.9 kg, (15 mg) -10.6 kg, (insulin glargine) +1.7 kg. Hypoglycemia less than 54 mg/dL in (5 mg) 8.8%, (10 mg) 6.1%, (15 mg) 8.0%, (insulin glargine) 19.1%. Fasting lipids change from precedent (15 mg) cholesterol -5.6%, triglycerides -22.5%, VLDL -21.8%, HDL +10.8%
SURPASS-5 [26] (NCT04039503)	N = 475, 40 weeks, double-blind	T2DM, 7.0% to 10.5% HbA1C, ≥3 months treatment of insulin glargine (U100) once daily with or without metformin, stable body weight ≥3 months, BMI of ≥23 kg/m2	Agents: weekly SC dose of 5 mg tirzepatide (n = 116), 10 mg tirzepatide (n = 119), 15 mg tirzepatide (n = 120); intervention: SC placebo (n = 120)	HbA1C mean change from precedent (5 mg) -2.11%, (10 mg) -2.40%, (15 mg) -2.34%, (placebo) -0.86%	Body weight changes from precedent (5 mg) -5.4 kg, (10 mg) -7.5 kg, (15 mg) -8.8 kg, (placebo) +1.6 kg. Hypoglycemia less than 54 mg/dL in (5 mg) 15.5%, (10 mg) 19.3%, (15 mg) 14.2%, (placebo) 12.5%
SURPASS J-mono [27] (NCT03861052)	N = 636, 52 weeks, double-blind	T2DM, HbA1C between 7.0% and 10.0% if OAM is naive, HbA1C = 6.5% to 9% if currently on OAM, BMI of ≥23 kg/m2, stable body weight ≥3 months with no exercise/intensive diet for body weight reduction	Agents: weekly SC dose of 5 mg tirzepatide, 10 mg tirzepatide, 15 mg tirzepatide; intervention: dulaglutide 0.75 mg	HbA1C mean change from precedent (5 mg) -2.37%, (10 mg) -2.55%, (15 mg) -2.82%, (dulaglutide) -1.29%	FSG changed from precedent (5 mg) -57.9 mg/dL, (10 mg) -64.6 mg/dL, (15 mg) -67.6 mg/dL, (dulaglutide) -31.9 mg/dL. Body weight changes from precedent (5 mg) -5.8 kg, (10 mg) -8.5 kg, (15 mg) -10.7 kg, (dulaglutide) -0.5 kg. Fasting insulin changes from precedent (5 mg) -1.07 mU/L, (10 mg) -1.87 mU/L, (15 mg) -2.00 mU/L, (dulaglutide) 1.4 mU/L. Fasting C-peptide change from precedent (5 mg) -0.25 ug/L, (10 mg) -0.39 ug/L, (15 mg) -0.37 ug/L, (dulaglutide) 0.01 ug/L
SURPASS J-combo [28] (NCT03861039)	N = 442, 52 weeks, open-label	T2DM, HbA1C between 7.0% and 11.0%, with ≥3 months metformin, sulfonylureas, thiazolidinediones, glinides, SGLT-2 inhibitor or alpha-glucosidase inhibitor, BMI of ≥23 kg/m2, stable body weight ≥3 months with no exercise/intensive diet for body weight reduction	Agents: weekly SC dose of 5 mg tirzepatide, 10 mg tirzepatide, 15 mg tirzepatide; intervention: oral antihyperglycemic medication (OAM)	≥1 SAE in patients	FSG changed from precedent (5 mg) -58.6 mg/dL, (10 mg) -71.2 mg/dL, (15 mg) -74.4 mg/dL. Body weight changes from precedent (5 mg) -3.8 kg, (10 mg) -7.5 kg, (15 mg) -10.2 kg. Fasting insulin changes from precedent (5 mg) 6.2 pmol/L, (10 mg) -4.8 pmol/L, (15 mg) -7.7 pmol/L. Fasting C-peptide change from precedent (5 mg) -0.12 ug/L, (10 mg) -0.28 ug/L, (15 mg) -0.34 ug/L
SURMOUNT-1 [29] (NCT04184622)	N = 2,539, 72 weeks, double-blind placebo-controlled	≥18 years old, BMI ≥ 30 kg/m², or ≥27 kg/m², and previous diagnosis with at least one of the following comorbidities: hypertension, dyslipidemia, obstructive sleep apnea, and cardiovascular disease. History of at least one unsuccessful dietary effort to lose body weight	Agents: weekly SC dose of 5 mg tirzepatide, 10 mg tirzepatide, 15 mg tirzepatide; intervention: weekly SC placebo	Reduction in body weight from precedent (5 mg) 15%, (10 mg) 19.5%, (15 mg) 20.9%, (placebo) 3.1%	Change in waist circumference from precedent (5 mg) -14.0 cm, (10 mg) -17.7 cm, (15 mg) -18.5 cm. Percentage weight reduction of ≥20% (5 mg, not controlled for type 1 error) 48%, (10 mg) 67%, and (15 mg) 71% (placebo) 9%
Completed trials without data available					
SURPASS-AP combo [30] (NCT04093752) Completion date: November 2021	N = 917, 40 weeks, open-label	T2DM, treatment with metformin with or without sulfonylurea for ≥2 months, HbA1C between 7.5% and 11.0%, BMI of ≥23 kg/m2, stable body weight ≥3 months with no exercise/intensive diet for body weight reduction	Agents: weekly SC dose of 5 mg tirzepatide, 10 mg tirzepatide, 15 mg tirzepatide; intervention: dose of insulin glargine	Mean HbA1C change from precedent	Not published

Multicentric and international clinical trials are the hallmark of the SURPASS phase III program [8-10,25,26], with SURPASS 1 [25] being conducted at 52 medical research institutes and hospitals in India, Japan, Mexico, and the United States. The SURPASS 2 [8] clinical studies enrolled participants from the United States, Argentina, Australia, Brazil, Canada, Israel, Mexico, and the United Kingdom. SURPASS 3 [9] was conducted across 45 sites in six countries (Hungary, Poland, Romania, Spain, Ukraine, and the USA), while SURPASS 4 [10] and SURPASS 5 [26] were conducted at 187 sites in 14 countries on five continents and 45 medical research centers and hospitals in eight countries, respectively; two studies [27,28] are from Japan, and one is from the Asia-Pacific region [30] with the recruitment of participants from Australia, China, India, and the Republic of Korea. In contrast to SURPASS 1 [25], which treated people who had never been treated before, other studies [8-10,26-28,30] dealt with patients who were taking a variety of oral anti-diabetic drugs, including insulin, metformin, sulfonylurea, pioglitazone, and/or sodium-glucose cotransporter 2 (SGLT-2) inhibitors. These include GLP-1 receptor agonists (GLP-1Ras) (dulaglutide and semaglutide) [8,27], long-acting insulin analogs (glargine and degludec) [9,10,30], or short-acting insulin analogs [28]. Both SURPASS 1 [25] and SURPASS 5 [26] were regulated by placebo.

The SURPASS trials looked at once-weekly tirzepatide doses of 5 mg, 10 mg, and 15 mg using a dose escalation algorithm. The starting dose was 2.5 mg weekly for the first four weeks, then 2.5 mg increased every four weeks until the maintenance level was attained. With the exception of the SURPASS J-combo trial, all studies used the change in HbA1c from baseline as their primary outcome [28]. Tirzepatide was found to be superior to other treatment options with a greater reduction in HbA1C in studies such as SURPASS 1-5 and J-mono [8-10,25-27].

Furthermore, considerable weight loss was observed in comparison to placebo, insulin, or semaglutide [31]. In the completed SURPASS J-combo [28] trial, tirzepatide has shown glycemic control improvement and a reduction in body weight regardless of the patient's underlying medications and is well tolerated as an adjunct to oral antihyperglycemic monotherapy in Japanese patients with T2DM. In the recently released SURMOUNT-1 [29] study, considerable weight reduction was observed in non-diabetics with all three doses of tirzepatide compared to placebo. This suggests that tirzepatide may be a promising treatment option for those living with obesity.

Prospective cardiometabolic application and cardiovascular implications of tirzepatide

Cardiometabolic disorders like diabetes mellitus, obesity, hypertension, heart disease, hyperlipidemia, and their vascular complications, including the likes of ischemic cardiomyopathy, stroke, neuropathy, retinopathy, and chronic kidney disease, are currently some of the leading causes of morbidity, disability, and mortality globally [32,33]. The alterations, especially at the cellular level, of the above-mentioned diseases occur years prior to the appearance of the characteristic clinical manifestations. The drug tirzepatide, which has the ability to enhance insulin sensitivity, lower blood glucose levels, decrease weight, and reform dyslipidemia, is deservedly considered to have the potential to be more than just an antidiabetic drug but also as a drug that can be potentially used in the management of above said morbidities. It is too early to be unduly optimistic, given that it is still necessary to assess the long-term effects of these substances and confirm their prospective cardiovascular advantages [32].

Role of GLP-1 and GIP in Cardiovascular Outcomes

Both GIP and GLP-1 have exhibited beneficial effects on various cardiovascular complications, thereby shedding light on their therapeutic potential over and above their potential to improve diabetic complications. Both receptors are expressed in the heart as well as in the blood vessels. The mRNA for GLP-1 receptors has been detected in all four chambers of the heart, and the GLP-1 receptor protein is significantly expressed in the sino-atrial node [34]. The GLP-1 receptor agonists have a wide range of beneficial effects as well as therapeutic potential concerning the cardiovascular system, especially via their inhibition of the development, progression, and rupture of atherosclerotic plaques, as proven by multiple studies [34,35]. It has been proven that any long-acting glucagon-like peptide-1 receptor (GLP-1R) agonist has anti-inflammatory properties similar to those of natural GLP-1 and can therefore help to protect organs like the heart and kidneys [36].

On the other hand, the GIP receptors in the heart have been assigned more of a functional role [34]. Even though information regarding the cardiovascular effects of GIP is scarcely available, a review by Heimbürger et al. has shed light on the various cardiovascular actions of GIP, including raising the heart rate, tissue-specific vasodilation, and blood flow regulation [34,37]. Animal studies have shown the anti-atherosclerotic activity of GIP. Furthermore, GIP is found to reduce oxidative stress in human endothelial cells and inflammatory cytokine release in visceral adipose tissue [34]. Pharmacological concentrations of GIP agonists are more likely to yield protective effects against atherosclerosis [36]. Nevertheless, further studies are required to obtain absolute clarity regarding the stimulation of GIP in the cardiovascular system [34].

Effect on Lipoprotein Biomarkers and Impact on Atherogenic Lipoprotein Profiles

The effectiveness and safety of tirzepatide were assessed in participants with T2DM that was not well managed by diet and exercise alone or stable metformin medication during a 26-week, double-blind, placebo-controlled, phase 2b trial. Participants in this phase 2b research were randomly assigned to receive a weekly subcutaneous injection of tirzepatide 1 mg, 5 mg, 10 mg, or 15 mg, 1.5 mg of the GLP-1R agonist dulaglutide, or a placebo for a duration of 26 weeks. According to the outcomes of this trial, higher doses of tirzepatide were associated with lower levels of total cholesterol after week 26 in participants with T2DM when compared to placebo. Higher doses of tirzepatide were also associated with lower levels of triglyceride when compared to placebo and dulaglutide. When compared to dulaglutide or the placebo, tirzepatide dose-dependently decreased triglyceride, serum lipoprotein profile, apolipoprotein (apo) C-III, and apo B levels over time. It also decreased the number of small low-density lipoprotein particles (LDLP) and large TAG-rich lipoprotein particles (TRLP). These findings may have consequences for cardiovascular safety because they point to a change in the lipoprotein profile of T2DM patients taking tirzepatide toward a less atherogenic one and a net improvement in insulin sensitivity [38].

Tirzepatide being a dual GLP-1 and GIP agonist, it is suggested that the GIP component of dual agonism may be responsible for the additional benefit, given the potential benefit of GIP receptor agonism on peripheral insulin sensitization. Consequently, tirzepatide was more effective than dulaglutide in reducing fasting dietary TAG [6,7]. Additionally, tirzepatide decreased atherogenic lipoprotein subclasses and lipoproteins containing apoB. For instance, smaller, dense LDLP and larger TAG-rich particles had lower fasting levels. In conclusion, the dose-dependent reduction in apoC-III and apoB levels, as well as the quantity of large TRLP and small LDLP, following tirzepatide therapy suggests a net improvement in the atherogenic lipoprotein profile [6,24,38].

Clinical Trials in Cardiovascular Safety

Tirzepatide shows positive effects on a variety of cardiovascular risk factors, but until now, the findings of just one study, SURPASS-4, have been used to report on the drug's cardiovascular safety [39]. The SURPASS-4 clinical trial evaluated tirzepatide versus insulin glargine in adults with T2DM and high cardiovascular risk who were not adequately controlled on oral glucose-lowering medications, with a particular emphasis placed on cardiovascular safety. This open-label, parallel-group, phase-three study, conducted in 187 sites in 14 countries across five continents, revealed that tirzepatide, when compared to glargine, demonstrated greater and clinically meaningful HbA1c and did not increase any cardiovascular risk [10].

Additionally, a prespecified meta-analysis on the tirzepatide cardiovascular event risk assessment was carried out for the SURPASS tirzepatide T2DM clinical development program. This prospectively planned, pooled individual participant data cardiovascular safety meta-analysis came to the conclusion that treatment with once-weekly tirzepatide at doses of 5 mg, 10 mg, and 15 mg, with controlled treatment exposure up to 104 weeks, was not linked to an increased risk for cardiovascular events in people with T2DM across a spectrum of T2DM duration and cardiovascular risk levels. Moreover, some of these findings are highly promising for tirzepatide's potential cardiovascular benefits, which are being examined in the current SURPASS-CVOT study [39].

Clinical significance

Tirzepatide has shown significant clinical promise as a potential dual-agonist therapy for diabetes, obesity, and related cardiometabolic disorders, and it may help address the urgent public health need for effective treatments, as highlighted by recent studies published by Kristensen et al. and Guh et al. [40,41]. By inducing an incretin effect that acts on pancreatic beta cells in an insulinotropic manner, tirzepatide effectively lowers glycemic levels and enhances insulin sensitivity [42]. There was a substantial reduction (−2.40% with 10-mg tirzepatide, −2.34% with 15-mg tirzepatide, and −0.86% with placebo) of HbA1c from the pretreatment baseline [26]. The glycemic efficacy of dual agonists improves insulin sensitivity, glucagon secretion, and beta cell function [43]. The SURPASS 1-4 clinical studies revealed an improvement in the tirzepatide-treated patients, who had HbA1c levels that were less than 5.7%, which is regarded as non-diabetic. The subgroup of patients who achieved different HbA1c targets (<5.7%, 5.7-6.5%, or 6.5%) is characterized by age, diabetic duration, a lower HbA1c, a fasting plasma glucose baseline, and glycemic and body weight reduction. BMI at baseline did not differ from that of those who did not achieve a normal HbA1c, indicating significant inter-individual variability in treatment effects, with a greater response in patients with a low duration of T2DM [44].

In terms of obesity, tirzepatide has shown promising results, which can be seen in the SURMOUNT-1 trial [29], where a substantial reduction in body weight was noted. Based on body mass, the assigned participants were categorized and received once-weekly, subcutaneous tirzepatide (5 mg, 10 mg, or 15 mg) or placebo for 72 weeks. At week 72, the average weight reduction was -15.0% with 5 mg, -19.5% with 10 mg, and -20.9% with a 15 mg weekly dose as compared to -3.1% with a placebo, which shows a significant degree of weight reduction.

Patients with T2DM experience a higher occurrence of non-alcoholic fatty liver disease (NAFLD), which can advance to hepatocellular carcinoma, cirrhosis, and liver failure. Additionally, there is a poorer correlation between NAFLD and acquiring T2DM [45]. Reducing body weight and using appropriate diet and exercise therapies are still considered superior treatments for NAFLD and NASH [46]. However, research on tirzepatide's effects on weight reduction suggests that it could be more effective for NASH patients. In a study conducted by Hartman et al. [47], individuals with T2DM received tirzepatide dosages that markedly reduced NASH-related biomarkers and raised adiponectin. Post hoc analyses demonstrated a significant decrease in aspartate aminotransferase (AST), serum alanine aminotransferase (ALT), and M30 fragment from baseline. Tirzepatide 10 mg and 15 mg caused higher reductions in ALT than dulaglutide, which enhanced procollagen III (pro-C3) and keratin-18 (K-18). K-18 fragments are useful for the diagnosis of NASH. During hepatocyte apoptosis, K-18 is cleaved, which elevates plasma levels of K-18 in NASH patients. Pro-C3 is a marker for fibrosis in T2DM patients. By week 26, 15 mg of tirzepatide led to a significant decrease in pro-C3. Thus, higher doses of tirzepatide showed improvement in NASH-related biomarkers. The results of this study cannot be interpreted as evidence of the effect of this therapy on NASH because of limitations such as the unequal distribution of patients between groups and incomplete data assessments. Therefore, a requirement for further evaluation is needed to support the findings of this study [47-49].

Comparatives effectiveness

Tirzepatide vs. Semaglutide

Semaglutide, also known as Ozempic or Wegovy, is a GLP-1 analog approved for the treatment of T2DM, cardiovascular risk reduction in patients with T2DM, and treatment of obesity [50-59]. Studies are being done on tirzepatide for similar claims. There are two studies [8,60] that compare tirzepatide and semaglutide and are relevant to our study. Frías et al. conducted the first clinical trial, in which patients received tirzepatide at doses of 5 mg, 10 mg, or 15 mg or semaglutide at a dose of 1 mg. The study's inclusion criteria were age 18 years or older and T2DM that had not been successfully controlled with metformin at a dose of at least 1500 mg per day. The study aimed to compare the safety and effectiveness of once-weekly tirzepatide to semaglutide [8]. The study's findings revealed that tirzepatide was not inferior to semaglutide but also superior (Table 2).

**Table 2:** Comparison between efficacy and safety of tirzepatide and semaglutide

Parameters	Tirzepatide - 5 mg, 10 mg, 15 mg	Semaglutide - 1 mg
Glycated hemoglobin level	>-2.01	-1.86
body weight reduction	More	Less
Nausea	17-22%	18%
Diarrhea	13-16%	12%
Vomiting	6-10%	8%
Hypoglycemic effect	More	Less
Serious adverse effect	5-7%	3%

The other study conducted by Vadher et al. found that tirzepatide 10 and 15 mg significantly reduced body weight and HbA1c compared to semaglutide 2 mg [61]. The major finding is that there are no significant differences observed between tirzepatide (5 mg) and semaglutide (2 mg) based on changes in baseline HbA1c levels and body weight. Hence, tirzepatide is a promising drug for reducing obesity and HbA1C levels when given at an appropriate dosage.

Tirzepatide vs. Insulin Degludec

SURPASS-3 [9] is a phase 3 study, with an inclusion criterion of age greater than 18 years, a baseline HbA1c of 7.0-10.5%, BMI of at least 25 kg/m2, and participants who were insulin-naive and treated with metformin alone or in combination with an SGLT-2 inhibitor for at least three months before screening. The participants were randomly injected with either tirzepatide (5 mg, 10 mg, or 15 mg) or titrated insulin degludec subcutaneously. At week 52, the HbA1c reductions were 1.93% for tirzepatide 5 mg, 2.20% (0.05) for tirzepatide 10 mg, and 2.37% for tirzepatide 15 mg, compared to a baseline of 1.34% with insulin degludec. Concluding that tirzepatide is superior to titrated insulin degludec in individuals with T2DM, resulting in higher drops in HbA1c and body weight at week 52 and a decreased risk of hypoglycemia.

Tirzepatide vs. Insulin Glargine

SURPASS-4 [10] was a phase 3 research study in which individuals were randomly allocated (1:1:1:3) and received a once-weekly subcutaneous injection of tirzepatide (5 mg, 10 mg, or 15 mg) or glargine (100 U/mL), titrated to achieve fasting blood glucose (FBG) levels less than 100 mg/dL. Tirzepatide 10 mg and 15 mg doses significantly improved HbA1c when compared to glargine.

Efficacy and safety

In terms of body weight, clinical trials found that tirzepatide considerably outperformed dulaglutide, semaglutide, degludec, and glargine in causing weight loss [7-10,62]. The likelihood that patients using tirzepatide would achieve an HbA1c of 5.7% was higher [9,10]. In addition, the positive effect on FBG and postprandial blood glucose (PPBG) is greater than that of dulaglutide, semaglutide, degludec, or glargine [8-10]. Tirzepatide showed a glucagonostatic action by lowering the level of glucagon, whereas separate injections of GIP or GLP-1 had no discernible impact [22,63,64].

Additionally, the waist circumference also decreased by 4.43 cm when using tirzepatide compared to semaglutide or dulaglutide and by roughly 4.83 cm when compared with a placebo [7,8,62]. The decrease in BMI was also comparatively substantial compared to dulaglutide, semaglutide, and placebo [22,63]. By week 40, the majority of patients in the tirzepatide group had significantly lower systolic and diastolic blood pressure than those in the placebo group (a difference of 6.1 to 12.6 mmHg and 1.7 to 2.1 mmHg, respectively) [8-10,25], and they also showed lower triglycerides and low-density lipoprotein cholesterol (LDL-C), and higher high-density lipoprotein cholesterol (HDL-C) as compared to other agents [65]. The side effects seen with tirzepatide were comparatively mild, with an overall low mortality rate. In all trials, a total of 41 deaths occurred (n = 4573) in the tirzepatide groups and 39 in the others (n = 2151), out of which 19 were related to coronavirus disease 2019 (COVID-19).

Ongoing trials

Tirzepatide is a strong weight-reduction and hypoglycemic medication, according to preclinical studies from phases 1 and 2 of SURPASS CVOT [66], with side effects comparable to those of GLP-1 receptor agonists. Tirzepatide's long-term effectiveness, safety, and cardiovascular risk are still being investigated in phase 3 trials [67]. The SURMOUNT-1 trial [29] studied the effects of tirzepatide on body weight in those who are overweight or obese by monitoring the change in baseline body weight, TAG, high-density lipoprotein, very-low-density lipoprotein, free fatty acids, etc. Another clinical trial (SYNERGY-NASH) is checking whether tirzepatide is safe and effective for the treatment of nonalcoholic steatohepatitis by monitoring the percentage of participants without NASH [66]. Long-term efficacy, safety, and cardiovascular risk of tirzepatide are presently being studied in phase 3 studies [67].

## Conclusions

Tirzepatide's simultaneous activation of GLP-1 and GIP receptors has proven significant therapeutic promise in multicentric and multinational phase III investigations. Comparative analysis of the SURPASS trials revealed that tirzepatide's glycemic efficacy and weight loss improved with dose in patients with T2DM when used as monotherapy and in combination with different antihyperglycemic drugs. In the SURPASS-4 study, tirzepatide exhibited a decrease in HbA1c levels of up to -2.58% from baseline values at the 15 mg dose. Similar studies using basal insulin and GLP-1 receptor antagonists produced statistically significant HbA1c control and body weight reduction, but no alteration in gastric side effects. Despite the limited data, tirzepatide has shown significant improvement in lipid profiles and cardiovascular and renal risks.

SURMOUNT-1, a randomized phase III study in which tirzepatide's efficacy was investigated in patients with overweight and obesity without T2DM, yielded promising results for the drug's prospective applications in obesity, heart failure, and NAFLD. The percentages of individuals who had a substantial weight reduction of 5% or more were 85%, 89%, and 91% with tirzepatide 5 mg, 10 mg, and 15 mg, respectively. In all the studies conducted, a reduction in BMI and waist circumference was evidenced, along with improvements in lipids, low-density lipoprotein, and triglycerides. With studies indicating that tirzepatide outperforms long-acting insulin in maintaining postprandial glucose levels, the gastrointestinal adverse effects of tirzepatide could be behind this phenomenon. T2DM is a highly morbid disease that necessitates a treatment that is not only confined to the effectiveness of the drug but also has fewer complications. Further research with larger datasets compiling psychiatric and economic variables alongside pharmacotherapeutic outcomes might concretize the novelty of tirzepatide.
